# CVRmap—a complete cerebrovascular reactivity mapping post-processing BIDS toolbox

**DOI:** 10.1038/s41598-024-57572-3

**Published:** 2024-03-27

**Authors:** A. Rovai, V. Lolli, N. Trotta, S. Goldman, X. De Tiège

**Affiliations:** 1 Université Libre de Bruxelles (ULB), ULB Neuroscience Institute (UNI), Laboratoire de Neuroanatomie et de Neuroimagerie translationnelles, Université libre de Bruxelles, Brussels, Belgium; 2 Université Libre de Bruxelles (ULB), Hôpital Universitaire de Bruxelles (HUB), CUB Hôpital Erasme, Department of Translational Neuroimaging, Université Libre de Bruxelles, Brussels, Belgium; 3 Université Libre de Bruxelles (ULB), Hôpital Universitaire de Bruxelles (HUB), Hôpital des Enfants Reine Fabiola, Department of Radiology, Université Libre de Bruxelles, Brussels, Belgium; 4 Université Libre de Bruxelles (ULB), Hôpital Universitaire de Bruxelles (HUB), CUB Hôpital Erasme, Department of Radiology, Université Libre de Bruxelles, Brussels, Belgium

**Keywords:** Functional magnetic resonance imaging, Neuro-vascular interactions, Neurophysiology

## Abstract

Cerebrovascular Reactivity (CVR) refers to the ability of cerebral blood vessels to dilate or constrict under the effect of vasoactive substances and can be estimated using functional Magnetic Resonance Imaging (fMRI). Computation of CVR maps is relevant in various brain diseases and requires specialized data processing. We introduce CVRmap, an opensource software that automates the computation of CVR map. The toolbox complies with the Brain Imaging Data Structure (BIDS) standards.

## Introduction

Cerebro-Vascular Reactivity (CVR) quantifies the ability of cerebral vessels to dilate or constrict under the effect of vasoactive substances such as the blood CO_2_ partial pressure. It can be estimated using functional Magnetic Resonance Imaging (fMRI) since the Blood Oxygen Level Dependent (BOLD) signal is a surrogate for local blood flow^1–3^, and blood CO_2_ partial pressure can be altered using breathing challenges such as CO_2_ inhalation^4^, breath-hold^5^, progressive hypercapnia^6^, end-tidal CO_2_ forcing^7^ or even resting-state^8^. The relative change in the BOLD signal is then divided by the change in CO_2_ blood partial pressure to obtain CVR^9,10^. CVR can be used to assess vascular integrity and has a growing range of clinical applications^11,12^. In most studies using CVR, preprocessing and CVR mapping is done using in-house scripts, posing several problems regarding results reproducibility and methodological transparency. Moreover, there are no openly accessible datasets on which CVR methods can be experienced, tested, or compared.

## Main

This work aims to fill these gaps by offering to the community a standard framework for the computation of CVR in a variety of research topic, whether in clinical or more neuroscience-oriented projects, as well as an openly accessible normative dataset. CVRmap complies with the Brain Imaging Data Structure (BIDS)^13^ standards for data processing applications. It works hand-in-hand with preprocessed data from fMRIprep^14^ but could also be used with other conventional preprocessing pipelines provided the BIDS layout is respected. With this effort, we push the field to be part of the openscience culture, fostering the transparency of the methods, collaborative work and data sharing.

**Figure 1:**
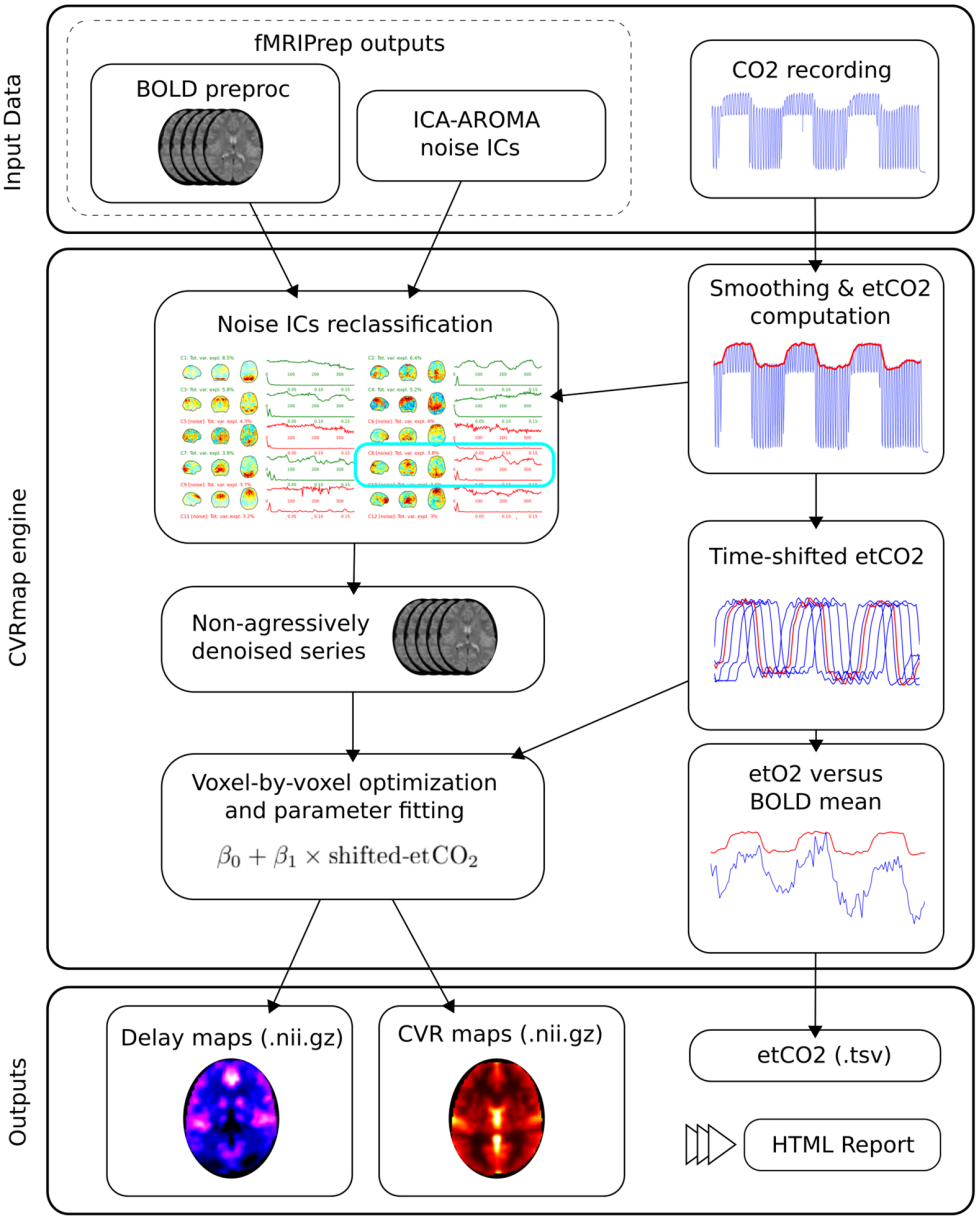
Overview of the CVRmap workflow. CVRmap runs on data preprocessed with fMRIPrep together with raw CO_2_ readings. The end-tidal CO_2_ timecourse is computed after a light smoothing. The noise sources as classified by ICA-AROMA are compared to the etCO_2_ to ensure physiogological signal of interest is not removed from the BOLD signal. The non-aggressively denoised data are then modeled with various time-shifted version of the etCO_2_ timecourse to determine optimum delay and compute CVR from the fitted parameters. The pipeline saves delay and CVR maps, etCO_2_ timecourse and a summary HTML report including control checkpoints.

**Figure 2:**
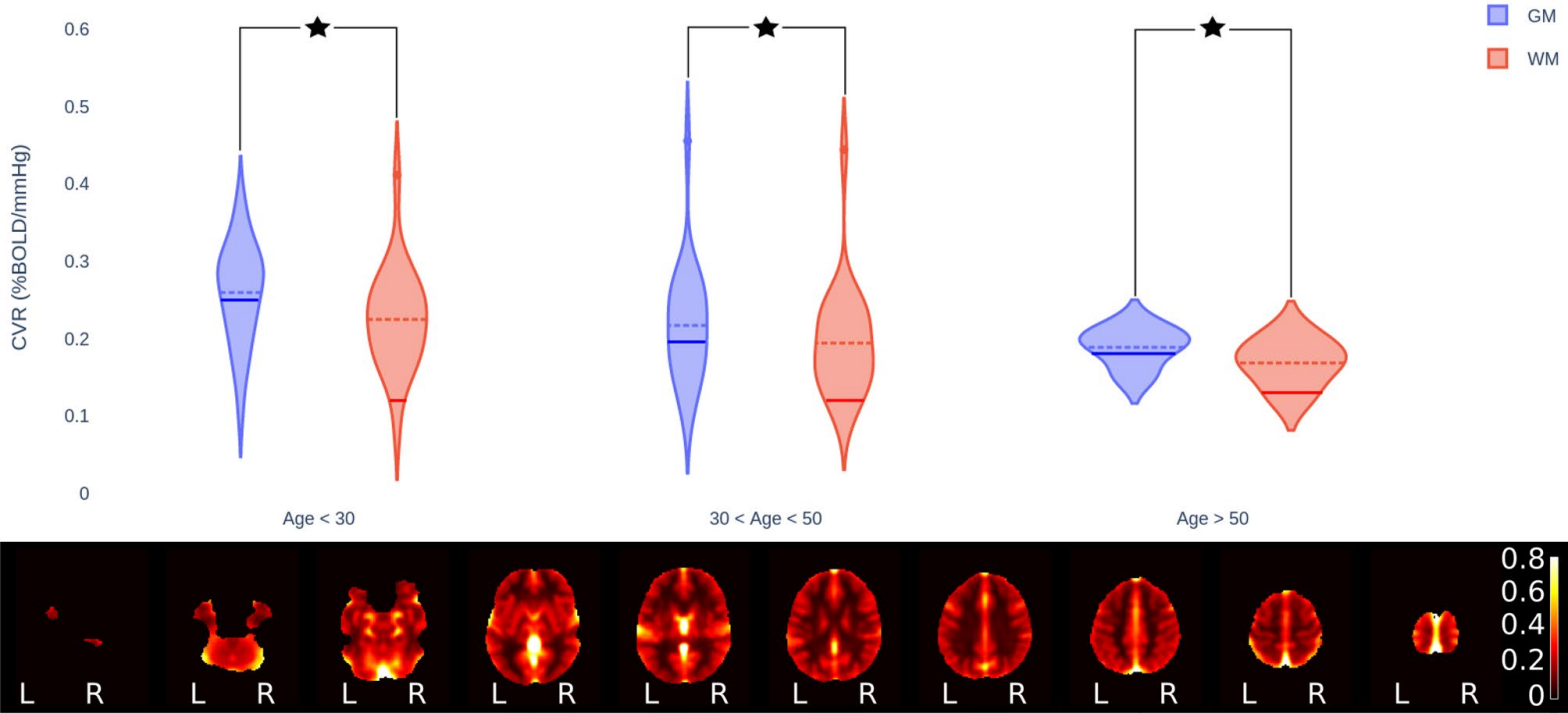
Results for the normative dataset. *Violin plots*: mean CVR for gray matter (GM) and white matter (WM), grouped by age. Dashed lines represent the mean of each distribution. Solid lines correspond to values found in the literature: for GM, it is taken from the previous study analyzing GM CVR decade-by-decade^21^ and for WM it is taken from^10^. A star indicate statistical significance at *p*-value less than $5\%$ for the corresponding paired *t*-tests. *Mosaic view of axial slices*: dataset mean of CVR maps in template (MNI) space, corrected for age (units are %BOLD/mmHg). Displayed using the neurological convention.

**Figure 3:**
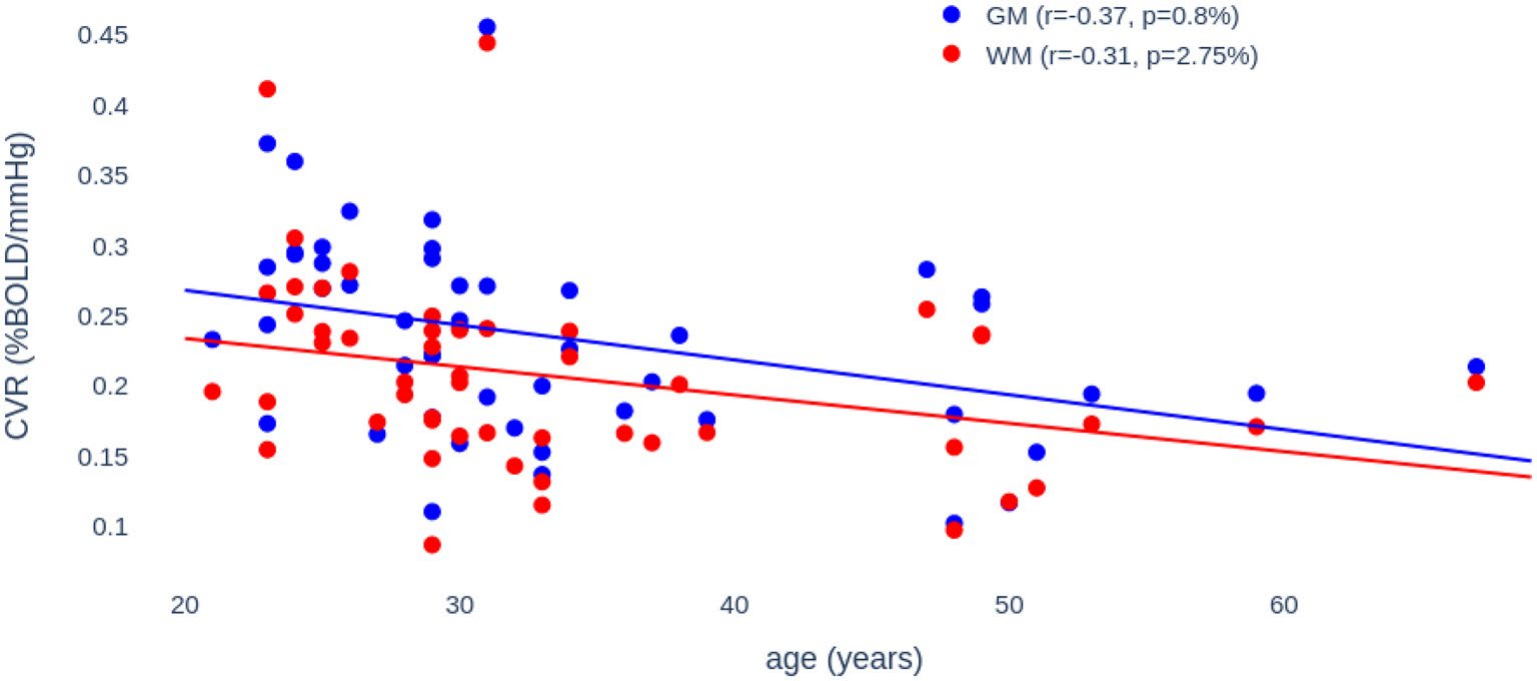
Individual Report are produced for each participant. The reports are saved in html format. Sections include a summary of the job and the following control checkpoints: recorded physiological data versus upper envelope (etCO_2_), Global BOLD signal versus etCO_2_ for alignment verification, and a representative set of axial slices, using neurological convention, of the delay and CVR maps. The report ends with a summary of the noise-IC re-classification described in the main text. Report structure has been reformatted for visual purposes.

**Figure 4:**
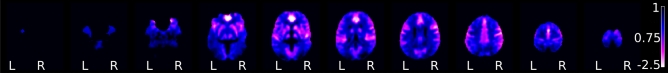
Effect of age on CVR for gray matter (GM) and white matter (WM). Data from the derivatives of CVRmap for 50 healthy participants acquired to validate the pipeline. The participants underwent CO_2_ inhalation challenges using the protocol described in^4^. The straight lines correspond to the fitted models.

**Figure 5:**
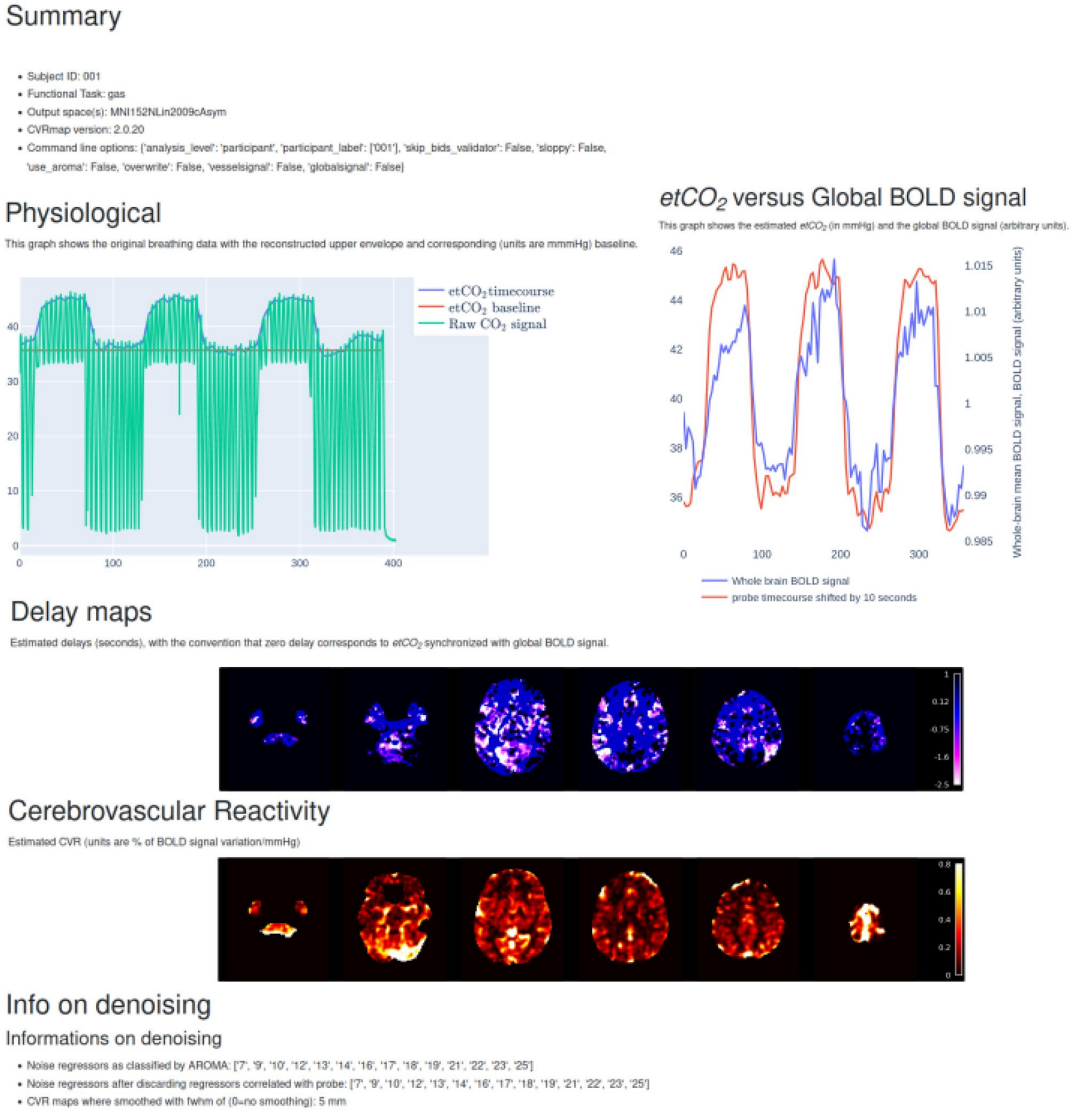
Mean of delay maps in normative dataset, in template (MNI) space and corrected for age (units: seconds). The delays are shifted as explained in the main text to set the global signal delay to zero. White and light purple-colored regions are reached before blue and darker ones. Displayed using the neurological convention.

Imaging data must be accompanied by recordings of breath CO_2_ concentration and converted into a BIDS-compliant dataset as described in the BIDS documentation. The user can launch the CVRmap pipeline after preprocessing by fMRIPrep. There is no other requirement on the data structure, making the stream stable and easy to use. The pipeline checks that the provided data matches the expected inputs and produces readable error messages if the dataset is not suitable for CVR analysis.

The pipeline is agnostic of the experimental paradigm: the mathematics used to model the BOLD signal and estimate CVR^9^ holds irrespective to the breathing challenge. Researchers can therefore analyze their data from different protocols using the same exact tool, allowing a reliable comparison of the results.

The first step is to estimate the CO_2_ blood partial pressure timecourse from the breathing CO_2_ recordings. A faithful proxy of blood CO_2_ is the end-tidal CO_2_ (etCO_2_), which corresponds to the upper envelope of the breathing data. The pipeline performs this physiological data preprocessing step automatically, removing breathing patterns and taking care of potential noise sources by smoothing the timecourse. These steps are compatible with virtually any CO_2_-recording device, in particular, the pipeline accommodates for any sampling frequency.

The second step is to denoise the fMRI data using the fMRIPrep outputs which includes Independent Component Analysis (ICA)-based strategy for Automatic Removal of Motion Artefacts (ICA-AROMA^15^). CVRmap uses a refinement of the ICA-AROMA classification to ensure that noisy Independent Component (IC) do not share resemblance with the CO_2_ proxy^16^. This re-selection of noise ICs is necessary to ensure that physiological signal of interest is not removed from the data. The non-aggressively denoised data emerging from this procedure are automatically computed^15^.

The final step deals with time delays expected between etCO_2_ and BOLD signal. The pipeline takes care of local time delays on a voxel-by-voxel basis by systematically exploring time shifts between etCO_2_ and BOLD signals^17–20^. This shift-and-search procedure implies a robust extrapolation of the etCO_2_ curve without the necessity of trimming the original BOLD signal. The results are summarized on delay maps, the time of reference being set to the global BOLD signal. The BOLD signal is modeled using the optimally shifted etCO_2_ at each voxel^9^:

$$\begin{aligned} \text {BOLD} = \beta _0 + \beta _1 \times \text {shifted-etCO}_2\,, \end{aligned}$$

where $\beta _0$ and $\beta _1$ are fitted parameters at optimal time-shifts, from which CVR is computed by

$$\begin{aligned} \text {CVR} = \frac{\beta _1}{\beta _0 + \beta _1 \times \text {etCO}_2\text {-baseline} }\, \cdot \end{aligned}$$

All these steps of the pipeline are summarized on the workflow diagram on Fig. 1.

Users have access to all intermediate steps and final results of the pipeline through easy-to-read and share visual reports for each participant. It includes original and preprocessed physiological and global BOLD signal timecourses for quality check purposes and present a summary of the noise-IC re-selection process. CVR and maps of optimal delay are presented using mosaic layouts. The report, being written in HTML, can be opened in any web-browser.

CVR and delay maps are saved in accordance with the BIDS derivatives standard as compressed NIFTI files in either participant or Montreal Neurological Institute (MNI) template space. The former is useful for patients, whose anatomical abnormalities may compromize the quality of the normalization to template space, and can be directly coregistered and overlayed with original anatomical scans. Maps in template space, derived from the sophisticated outputs of fMRIPrep, can be used to perform group-level analysis. CVRmap is limited to participant-level processing, but the easy and intuitive layout of the outputs, following the guidelines of the BIDS for derived data, makes it easy to incorporate the individual results into custom group-level analysis pipelines or any other region-of-interest analysis.

The pipeline was validated on a cohort of 50 healthy adults (25 females, mean age: 33.6 y, age range 21–67 y) who underwent an fMRI session with hypercapnic blocs following the protocol of^4^. CVRmap finished without errors on the whole dataset. Mean CVR was computed in gray matter (GM) and white matter (WM) and grouped in three age categories (less than 30 y, between 30y and 50 y, and more than 50 y). The results are presented on Fig. 2, and show a higher CVR in GM than in WM for each age group and a decrease of CVR with age (in agreement with the data from the literature^10,21^). We also show mean CVR maps in axial plane in MNI space, obtained by fitting a General Linear Model with the age of the participant as a regressor to extract age-corrected data. The dataset is shared online, allowing researchers to perform quantitative comparison between their data and this normative dataset.

## Methods

CVRmap is an opensource software that computes maps of Cerebro-Vascular Reactivity from fMRI data recorded alongside continuous breathing CO_2_ measurements. It is developed in the powerful framework of the BIDS^13^, in particular taking advantage of the robust preprocessing tool fMRIPrep^22^. CVRmap has various installation solutions including containerized environments, which makes it easy to use by clinicians or researchers without advanced skills in programming. More specifically, basic knowledge of the command-line in Unix-like environment is the only prerequisite to use CVRmap. It runs mostly automatically, and yet can be adapted to the user’s specific needs owing to several run options such as participant label, target space, task name, denoising strategy, fMRIPrep and output directory. More specific needs can also by satisfied by specifying a custom configuration file to tune the parameters such as spatial or temporal smoothing strength. The processing for a single participant takes about 15 min to complete on a standard workstation (4 Gb of memory, Intel Core i7, using one thread). The comprehensive reports allow for a quick and efficient visual overview of the results. We refer to Fig. 3 for an example of such report.

The pipeline is agnostic of the experimental paradigm. For any breathing challenge allowing continuous monitoring of breathing CO_2_, e.g. CO_2_ inhalation, progressive hypercapnia^6^, or using end-tidal CO_2_ forcing devices^7^, the mathematics implemented in CVRmap and used to model the BOLD signal to eventually estimate CVR hold^9,23^. Researchers can therefore analyze their data from different protocols using the same exact tool, allowing a reliable comparison of the results. Moreover, the code is shared using a standard version-control system, giving full access to previous releases or single contributions to the package. It is also open to further contribution, possibly enlarging its field of applications.

### Input data

*fMRI data.* The rigidity of the BIDS standard allows the pipeline to adapt to different types of input data, such as sessions, runs, or custom acquisition labels. The raw BOLD series itself is not mandatory, since CVRmap works on data preprocessed by fMRIPrep. The only condition is that fMRIPrep produced ICA-AROMA classification of noise sources. CVR maps can be computed for any available space in the fMRIPrep derivatives, including participant’s space. By default, CVRmap will load any series matching the ‘gas’ taskname, but this can be customized to meet user’s needs.

Note that the details of the acquisition of the BOLD data, such as slice ordering or options like single-echo, multi-echo or multiband Echo Planar Imaging (EPI), are handled by fMRIPrep. In particular, preprocessing steps like slice-timing and realignment are automatically performed, setting the outputs of fMRIPrep on an equal footing for each of these acquisition schemes. Multi-echo EPI, whose relevance in the context of breath-hold CVR has been discussed in^16,24,25^, is automatically processed using the current state-of-the-art multi-echo denoising software *tedana*^26^ within fMRIPrep. For these reasons, CVRmap itself should be blind to these details and should run equally well for data acquired using any of these techniques.

*Physiological data.* Continuous recording of breath CO_2_ must be included in the dataset, using the file naming scheme imposed by the BIDS and matching the BOLD task entity. The metadata file must contain the CO_2_ sampling frequency and corresponding units. The pipeline will adapt any time-domain processing to any sampling frequency, ensuring compatibility with CO_2_ acquisition parameters. It includes consistency checks of the input data and produces readable error messages if the dataset is not suitable for CVR processing. More specifically, CVRmap checks that the BIDS dataset contains the required preprocessed data from fMRIPrep as well as the physiological recordings associated with the selected task name. If the breathing recording is shorter in duration than the BOLD data, the associated etCO_2_ trace is padded using a baseline interpolation. Further checks require visual inspection of the report (for instance for alignment verification between etCO_2_ and global BOLD signal).

### ﻿Physiological data preprocessing and quality check

Recordings of CO_2_ typically include uninteresting breathing patterns. A reliable proxy of blood CO_2_ partial pressure is the etCO_2_, which corresponds to the upper envelope of the breathing data. The pipeline performs this physiological data preprocessing step automatically, removing breathing patterns and taking care of potential noise contributions by smoothing the timecourse. The result is saved together with summary plots that can be used for quality check or illustrative purposes.

### Custom BOLD denoising

fMRI data are non-aggressively denoised within CVRmap using a refined version of the ICs-based classification of motion artefact ICA-AROMA^15^. The original classification is known to be suited for resting-state, task, or event-based paradigms, in which physiological sources of signal are considered to be noise. In the context of CVR mapping, this is not appropriate because CVR is of physiological origin^16^. To solve this, CVRmap automatically computes the linear correlation between the etCO_2_ timecourse and each noise ICs found by ICA-AROMA. Using an empirical threshold (the default value is set to 0.6, which is conventionally considered to be a moderate correlation. This can be changed by the user) on the computed correlation coefficient, CVRmap decides whether the IC should remain classified as noise or not. Based on this new classification, the denoised BOLD series are computed using the non-aggressive denoising scheme. The user can optionally adjust the threshold manually or even skip this step overall.

### Delay optimization and CVR processing

An important feature of CVR estimation is to include time delays in the analysis, which are of two types^5,10,27^:

1. *Global delay*: depending on the experimental setup, the CO_2_ concentration can be recorded with a delay with respect to the BOLD series. This delay depends for instance on the length of the sampling line extending from the participant’s mouth to the capnograph, typically located outside of the scanning room.
2. *Local delays*: the fluctuations of blood CO_2_ will not reach all brain regions simultaneously. As a consequence, the BOLD response cannot be expected to be synchronous in all brain regions.

CVRmap solves both these problems by computing time-shifted versions of the etCO_2_ timecourses^28^. This procedure implies a robust baseline extrapolation of the etCO_2_ curve without the necessity of trimming the original BOLD signal. The reference delay is set to the time-shift maximizing the correlation with the whole-brain BOLD mean signal. With this choice, any global delay from different experimental protocols is effectively eliminated, and does not rely on brain segmentation or parcellation. To find local delays, CVRmap finds the optimum time-shift by modeling the BOLD signal as a general linear model (GLM) at each voxel as $Y = \beta _0 + \beta _1 \times \text {shifted-etCO}_2$, saving the fitted parameters of the GLM at optimal time shift^28^. As a by-product of this procedure the pipeline computes CVR voxelwise using^9,23^

$$\begin{aligned} \text {CVR} = \frac{\beta _1}{\beta _0 + \beta _1 \times \text {etCO}_2\text {-baseline} }\,, \end{aligned}$$

using the estimated parameters $\beta _0$ and $\beta _1$ at optimal time-shift.

### Output maps

CVR and delay maps are saved in the space selected by the user and in accordance with the BIDS derivatives standard as compressed NIFTI files, allowing any researcher to use them for region-of-interest or statistical group analysis. Maps in participant space are particularly useful for patients, whose potential anatomical abnormalities can compromize the quality of the normalization to template space. They can be directly overlayed with original anatomical scans. CVRmap is limited to participant-level processing, but the easy and intuitive layout of the outputs, following the guidelines of the BIDS for derived data, makes it easy to incorporate the individual results into in-house pipelines.

### CVRmap report

Users have access to all intermediate steps and a summary of the final results of the pipeline through easy-to-read and share visual reports for each participant. It includes original and preprocessed physiological and global BOLD signal timecourses for quality check purposes and present a summary of the noise-IC re-selection process. CVR and maps of optimal delay are presented as mosaics of representative axial slices. The report, being written in HTML, can be opened in any web-browser and straightforwardly shared between peers or directly uploaded online.

## Open dataset and validation

The pipeline was validated on a cohort of 50 healthy adults (25 females, mean age: 33.6 y, age range 21–67 y) who underwent an fMRI session with hypercapnic blocs following a CO_2_ inhalation protocol as in^4^. The CO_2_ concentration was recorded at the level of their mouth using SmartLab Data Acquisition System (Hans Rudolf, Kansas, USA). The global delay between the BOLD and CO_2_ signals was approximately 10 s due to the length of the sampling line (6 meters). The full description of this dataset can be found in the Supplementary Information.

After BIDS conversion of the MRI data, preprocessing was done using fMRIPrep with ICA-AROMA flag and no other option. Physiological data from the SmartLab system where formatted according to the BIDS before launching CVRmap. The pipeline finished without errors on the whole dataset and each individual reports have been visually inspected to ensure correct gas delivery and adequate etCO_2_ timecourse estimation.

Two well-established features of CVR have been assessed on our dataset: (i) For a each subject, CVR in GM is larger than in WM and (ii) CVR in GM and WM both decrease with age. The method to extract the GM and WM masks is described in the fMRIPrep methods and is given as Supplementary Materials. The violin plot on Fig. 2 shows the CVR data from CVRmap grouped by age and are consistent with the literature^10,21^.

For each age group, mean CVR in GM was compared to mean CVR in WM using two-sample paired *t*-tests. In the three age groups, GM had significantly higher mean CVR than WM (all *p*-values lower than 0.001 %). As a complementary check, we linearly regressed GM and WM CVR means as a function of age (without grouping) and performed one-sample *t*-test on the fitted coefficients, see Fig. 4. For GM, the *r*-value $r_\text {GM} = -0.37$ and the *p*-value $p_\text {GM} = 0.8\%$; for WM, we found $r_\text {WM} = -0.31$ and $p_\text {WM} = 2.75\%$; we can therefore conclude that both these effects are significant at the conventional 5% *p*-value threshold.

For completeness, we also show on Fig. 5 the mean map of computed delays, which shows that GM is reached before WM, consistently with the literature^10^.

The dataset including raw data and derivatives is freely available online and can be used to test or compare other CVR computation methods, or can be used as a normative set to compare CVR of patients in research or clinical setups (Fig. 5).

### Comparison to other existing softwares

We have selected two other softwares to compare CVRmap: SeeVR^29,30^ and MRICloud^31^. SeeVR is opensource and written in MATLAB. It contains a wide range of tools to handle, explore and analyze CVR or, more generally, various hemodynamic aspects of fMRI data. Thanks to tutorial scripts, users can create their own pipelines to explore their data and optimize each step of the processing. It is designed to work with physiological recordings from a RespirAct system (Thornhill Medical, Toronto, Canada). MRICloud is a cloud-based service on which users can upload their data. The pipeline is described in^31^ but the exact implementation is not openly accessible. Apart from the anatomical and BOLD scan, there is no need (nor any possibility) to specify other parameters. Processing times are similar for the three softwares.

Both extremes come with their own benefits and disadvantages: the cost of SeeVR flexibility is that users need to have good scripting abilities to write their pipelines, but allow for potentially much more analyzes than CVRmap. MRICloud is very user-friendly but there is absolutely no freedom nor control on the processing. Moreover, as a web-based tool, its availability to users depends on the operational status of the servers and the associated computing clusters.

A summary of the distinctive features of SeeVR, MRICLoud and CVRmap is presented in Table 1.

**Table 1:** Comparison of CVRmap to SeeVR and MRICloud.

	SeeVR	MRICloud	CVRmap
BIDS application	No	No	Yes
Opensource	Yes	No	Yes
Requires scripting	Yes	No	No
License	GPL3	Closed source	AGPL3
Containerized solution	No	No	Yes
Spatial smoothing	Optional	8 mm FWHM	Optional
Denoising	Flexible	Motion parameters	ICA-AROMA based
Graphical User Interface	No	Yes (web-based)	No

### Summary

CVRmap is a specialized fMRI data processing program to compute maps of Cerebrovascular Reactivity complying with the BIDS standards. By sharing the code as well as a normative dataset of healthy participants, our goal is to offer to the community a standard framework for the study of CVR in potentially any relevant research topic, whether in clinical or more neuroscience-oriented projects. With this effort, we push the field to be part of the openscience culture, fostering the transparency of the methods, collaborative work and data sharing. Having such tools appears to be crucial given the quickly increasing applications of CVR.

### Ethical approval

The research protocol was approved by the CUB Hôpital Erasme Ethics Committee. Written informed consent was obtained from all participants prior to their participation. They were compensated monetarily for the loss of working hours and travel expenses.

### Supplementary Information

Supplementary Information.

## Data Availability

Raw, preprocessed and processed data from the normative dataset are available on openneuro^32^ under the Creative Commons license. All anatomical scans have been defaced using pydeface^33^.

## Code availability

CVRmap is available under the AGPLv3 license at https://github.com/ln2t/cvrmap. Docker images corresponding to every new release are made available on Docker Hub. The GitHub repository also contain instructions to preprocess the data using fMRIPrep providing sample commands to be adapted for any dataset. Commands used to analyze the data are included in the readme file. The code, used to generate the figures in the present paper from the openly accessible dataset, is shared in the form of a jupyter notebook at https://github.com/ln2t/cvrmap-paper. This script also serves as an example of how a group-level analysis can be easily designed from the processed individual data, and contain the two-sample paired *t*-tests comparing CVR in GM and WM in each age group. The data denoising included in CVRmap uses tools from the FMRIB Software Library (FSL, Release 6.0 (c) 2018, The University of Oxford). The CVRmap version used to process the data presented in this manuscript is 2.0.19, and fMRIPrep preprocessing was done using v21.0.4.
