# Supplementary material for: CVRmap—a complete cerebrovascular reactivity mapping post-processing BIDS toolbox
Source: Sci Rep. 2024 Mar 27;14:7252. doi: 10.1038/s41598-024-57572-3 (PMC10973431; doi:10.1038/s41598-024-57572-3)
Supplement: Supplementary file 1 — Supplementary Information. [file 41598_2024_57572_MOESM1_ESM.pdf]

Normative dataset description

Subjects. Fifty healthy adult subjects (25 females, mean age: 33.6y, age range 21-67y, standard deviation 10.5y) with no prior history of neurological or psychiatric disorder, cranial surgery or pulmonary diseases were included in this study. To eliminate potential effects of caffeine on BOLD signal and CVR, we asked the participants to avoid caffeine during the 4 hours preceding scanning.

MRI acquisition. All MRI data acquisitions were performed on a research-dedicated hybrid 3T SIGNA PET-MR scanner (GE Healthcare, Milwaukee, Wisconsin, USA) using a 24-channel head and neck coil. Subjects were comfortably placed in the supine position on the PET-MR bed. Their head was immobilized using soft padding. Earplugs and headphones were used to minimize the noise associated with MRI data acquisition.

Functional data were acquired by using single-shot Gradient-Echo EPI T2*-weighted images, covering the whole brain (time of repetition (TR)/time of echo (TE)/flip angle (α): 3000 msec/34 msec/90°; field of view: 28 cm; acquired matrix: 96 × 96; slice thickness: 3 mm; in-plane resolution: 2.9 × 2.9 mm; 40 slices; 120 volumes). Four dummy scans (total duration: 12 seconds) were obtained prior to each session to allow the MR signal to reach a steady state, and were subsequently automatically discarded by the scanner.

A 3D T1-weighted BRAVO sequence covering the whole brain was acquired to coregister the functional data on morphological data (TR/TE/α: 8.3 msec /3.1 msec/12°; isotropic voxels of 1 mm).

Gas delivery and recordings. Gas type and delivery was done following the guidelines given in [1]. More specifically, we used carbogen (5% of CO_2, 21% of O2 and 74% of N2) placed in a 200L Douglas bag. The bag was connected to a three-way valve that can be manually operated to allow room air or carbogen to flow in the breathing circuit. An operator stayed inside the scanner room to switch the valve according to the experimental paradigm. From the three-way valve, the system was connected through a gas delivery tube to a T-shaped two-way non-rebreathing valve (Hans Rudolf, 2700 series, Kansas, USA) to prevent exhaled air from being sent back into the Douglas bag. A sampling line for CO_2 recording was then connected to the mouth piece through a disposable bacteria and virus filter (AirLife uni-filter by CareFusion, Vermon Hills, IL, USA). An extra pair of tubes where also connected for raw flow estimation through differential pressure measurements.

Outside the scanning room, the CO_2 sampling line was connected to a Nomoline sampling tube and eventually to a sidestream CO_2 sensor (Masimo Corporation, Irvine CA, USA). The CO_2 and raw flow data were acquired using a SmartLab Data Acquisition System (Hans Rudolf, Kansas, USA) connected to a computer. The length of the raw flow dual tubes and the CO_2 sampling lines was 6 meters; both were recorded with a sampling frequency of 100 Hz. Recordings started in synchronization with the first valid fMRI volumes (after the dummy scans) and was stopped about 30 seconds after the end of the fMRI acquisition to allow the complete wash-out of the CO_2 sampling line.

Experimental paradigm. We opted for a 60-second block design, starting with hypercapnia, to allow for the recording of the CO_2 bolus wash-out during the last minute of acquisition. The data are therefore composed of 20 volumes of hypercapnia, followed by 20 volumes of normocapnia; the cycle being repeated three times for a total duration of 6 minutes. The participants were asked to breathe normally through their mouth without any other specific instructions. When possible or required by the subject, a nose clip was used to ensure correct mouth breathing. The raw flow live readings were used to ensure that the subjects continuously used they mouth to breathe during acquisition. In a similar fashion, the CO_2 has been monitored to ensure correct carbogen delivery.
Methodological Compliance. All methods used in this study are performed in accordance with the guidelines provided by the of the CUB Hôpital Erasme Ethics Committee as well as the regulations of the CUB Hôpital Erasme hospital.

fMRIPrep methods

Results included in this manuscript come from preprocessing performed using *fMRIPrep* 21.0.4 (Esteban et al. [2]; Esteban et al. [3]; RRID:SCR_016216), which is based on *Nipype* 1.6.1 (Gorgolewski et al. [4]; Gorgolewski et al. [5]; RRID:SCR_002502).

Anatomical data preprocessing A total of 1 T1-weighted (T1w) images were found within the input BIDS dataset.The T1-weighted (T1w) image was corrected for intensity non-uniformity (INU) with `N4BiasFieldCorrection` [6], distributed with ANTs 2.3.3 [7, RRID:SCR_004757], and used as T1w-reference throughout the workflow. The T1w-reference was then skull-stripped with a *Nipype* implementation of the `antsBrainExtraction.sh` workflow (from ANTs), using OASIS30ANTs as target template. Brain tissue

segmentation of cerebrospinal fluid (CSF), white-matter (WM) and gray-matter (GM) was performed on the brain-extracted T1w using `fast` [FSL 6.0.5.1:57b01774, RRID:SCR_002823, 8]. Brain surfaces were reconstructed using `recon-all` [FreeSurfer 6.0.1, RRID:SCR_001847, 9], and the brain mask estimated previously was refined with a custom variation of the method to reconcile ANTs-derived and FreeSurfer-derived segmentations of the cortical gray-matter of Mindboggle [RRID:SCR_002438, 10]. Volume-based spatial normalization to two standard spaces (MNI152NLin2009cAsym, MNI152NLin6Asym) was performed through nonlinear registration with `antsRegistration` (ANTs 2.3.3), using brain-extracted versions of both T1w reference and the T1w template. The following templates were selected for spatial normalization: *ICBM 152 Nonlinear Asymmetrical template version 2009c* [Fonov et al. [11], RRID:SCR_008796; TemplateFlow ID: MNI152NLin2009cAsym], *FSL's MNI ICBM 152 non-linear 6th Generation Asymmetric Average Brain Stereotaxic Registration Model* [Evans et al. [12], RRID:SCR_002823; TemplateFlow ID: MNI152NLin6Asym].

Functional data preprocessing For each of the 1 BOLD runs found per subject (across all tasks and sessions), the following preprocessing was performed. First, a reference volume and its skull-stripped version were generated using a custom methodology of *fMRIPrep*. Head-motion parameters with respect to the BOLD reference (transformation matrices, and six corresponding rotation and translation parameters) are estimated before any spatiotemporal filtering using `mcflirt` [FSL 6.0.5.1:57b01774, 13]. BOLD runs were slice-time corrected to 1.46s (0.5 of slice acquisition range 0s-2.92s) using `3dTshift` from AFNI [14, RRID:SCR_005927]. The BOLD time-series (including slice-timing correction when applied) were resampled onto their original, native space by applying the transforms to correct for head-motion. These resampled BOLD time-series will be referred to as *preprocessed BOLD in original space*, or just *preprocessed BOLD*. The BOLD reference was then co-registered to the T1w reference using `bbregister` (FreeSurfer) which implements boundary-based registration [15]. Co-registration was configured with six degrees of freedom. Several confounding time-series were calculated based on the *preprocessed BOLD*: framewise displacement (FD), DVARS and three region-wise global signals. FD was computed using two formulations following Power (absolute sum of relative motions, Power et al. [16]) and Jenkinson (relative root mean square displacement between affines, Jenkinson et al. [13]). FD and DVARS are calculated for each functional run, both using their implementations in *Nipype* [following the definitions by 16]. The three global signals are extracted within the CSF, the WM, and the whole-brain masks. Additionally, a set of physiological regressors were extracted to allow for component-based noise correction [*CompCor*, 17]. Principal components are estimated after high-pass filtering the *preprocessed BOLD* time-series (using a discrete cosine filter with 128s cut-off) for the two *CompCor* variants: temporal (tCompCor) and anatomical (aCompCor). tCompCor components are then calculated from the top 2% variable voxels within the brain mask. For aCompCor, three probabilistic masks (CSF, WM and combined CSF+WM) are generated in anatomical space. The implementation differs from that of Behzadi et al. in that instead of eroding the masks by 2 pixels on BOLD space, the aCompCor masks are subtracted a mask of pixels that likely contain a volume fraction of GM. This mask is obtained by dilating a GM mask extracted from the FreeSurfer's *aseg* segmentation, and it ensures components are not extracted from voxels containing a minimal fraction of GM. Finally, these masks are resampled into BOLD space and binarized by thresholding at 0.99 (as in the original implementation). Components are also calculated separately within the WM and CSF masks. For each CompCor decomposition, the k components with the largest singular values are retained, such that the retained components' time series are sufficient to explain 50 percent of variance across the nuisance mask (CSF, WM, combined, or temporal). The remaining components are dropped from consideration. The head-motion estimates calculated in the correction step were also placed within the corresponding confounds file. The confound time series derived from head motion estimates and global signals were expanded with the inclusion of temporal derivatives and quadratic terms for each [18]. Frames that exceeded a threshold of 0.5 mm FD or 1.5 standardised DVARS were annotated as motion outliers. The BOLD time-series were resampled into standard space, generating a *preprocessed BOLD run in MNI152NLin2009cAsym space*. First, a reference volume and its skull-stripped version were generated using a custom methodology of *fMRIPrep*. Automatic removal of motion artifacts using independent component analysis [ICA-AROMA, 19] was performed on the *preprocessed BOLD on MNI space* time-series after removal of non-steady state volumes and spatial smoothing with an isotropic, Gaussian kernel of 6mm FWHM (full-width half-maximum). Corresponding "non-aggresively" denoised runs were produced after such smoothing. Additionally, the "aggressive" noise-regressors were collected and placed in the corresponding confounds file. All resamplings can be performed with *a single interpolation step* by composing all the pertinent transformations (i.e. head-motion transform matrices, susceptibility distortion correction when available, and co-registrations to anatomical and output spaces). Gridded (volumetric) resamplings were performed using `antsApplyTransforms` (ANTs), configured

with Lanczos interpolation to minimize the smoothing effects of other kernels [20]. Non-gridded (surface) resamplings were performed using `mri_vol2surf` (FreeSurfer).

Many internal operations of *fMRIPrep* use *Nilearn* 0.8.1 [21, RRID:SCR_001362], mostly within the functional processing workflow. For more details of the pipeline, see the section corresponding to workflows in *fMRIPrep*'s documentation.

Copyright Waiver

The above boilerplate text was automatically generated by fMRIPrep with the express intention that users should copy and paste this text into their manuscripts *unchanged*. It is released under the CC0 license.

References

[1] Hanzhang Lu, Peiying Liu, Uma Yezhuvath, Yamei Cheng, Olga Marshall, and Yulin Ge. MRI Mapping of Cerebrovascular Reactivity via Gas Inhalation Challenges. *Journal of Visualized Experiments*, (94): 52306, December 2014. ISSN 1940-087X. doi: 10.3791/52306. URL http://www.jove.com/video/52306/mri-mapping-cerebrovascular-reactivity-via-gas-inhalation. 0/0.

[2] Oscar Esteban, Christopher Markiewicz, Ross W Blair, Craig Moodie, Ayse Ilkay Isik, Asier Erramuzpe Aliaga, James Kent, Mathias Goncalves, Elizabeth DuPre, Madeleine Snyder, Hiroyuki Oya, Satrajit Ghosh, Jessey Wright, Joke Durnez, Russell Poldrack, and Krzysztof Jacek Gorgolewski. fMRIPrep: a robust preprocessing pipeline for functional MRI. *Nature Methods*, 2018. doi: 10.1038/s41592-018-0235-4.

[3] Oscar Esteban, Ross Blair, Christopher J. Markiewicz, Shoshana L. Berleant, Craig Moodie, Feilong Ma, Ayse Ilkay Isik, Asier Erramuzpe, Mathias Kent, James D. andGoncalves, Elizabeth DuPre, Kevin R. Sitek, Daniel E. P. Gomez, Daniel J. Lurie, Zhifang Ye, Russell A. Poldrack, and Krzysztof J. Gorgolewski. fmriprep. *Software*, 2018. doi: 10.5281/zenodo.852659.

[4] K. Gorgolewski, C. D. Burns, C. Madison, D. Clark, Y. O. Halchenko, M. L. Waskom, and S. Ghosh. Nipype: a flexible, lightweight and extensible neuroimaging data processing framework in python. *Frontiers in Neuroinformatics*, 5:13, 2011. doi: 10.3389/fninf.2011.00013.

[5] Krzysztof J. Gorgolewski, Oscar Esteban, Christopher J. Markiewicz, Erik Ziegler, David Gage Ellis, Michael Philipp Notter, Dorota Jarecka, Hans Johnson, Christopher Burns, Alexandre Manhães-Savio, Carlo Hamalainen, Benjamin Yvernault, Taylor Salo, Kesshi Jordan, Mathias Goncalves, Michael Waskom, Daniel Clark, Jason Wong, Fred Loney, Marc Modat, Blake E Dewey, Cindee Madison, Matteo Visconti di Oleggio Castello, Michael G. Clark, Michael Dayan, Dav Clark, Anisha Keshavan, Basile Pinsard, Alexandre Gramfort, Shoshana Berleant, Dylan M. Nielson, Salma Bougacha, Gael Varoquaux, Ben Cipollini, Ross Markello, Ariel Rokem, Brendan Moloney, Yaroslav O. Halchenko, Demian Wassermann, Michael Hanke, Christian Horea, Jakub Kaczmarzyk, Gilles de Hollander, Elizabeth DuPre, Ashley Gillman, David Mordom, Colin Buchanan, Rosalia Tungaraza, Wolfgang M. Pauli, Shariq Iqbal, Sharad Sikka, Matteo Mancini, Yannick Schwartz, Ian B. Malone, Mathieu Dubois, Caroline Frohlich, David Welch, Jessica Forbes, James Kent, Aimi Watanabe, Chad Cumba, Julia M. Huntenburg, Erik Kastman, B. Nolan Nichols, Arman Eshaghi, Daniel Ginsburg, Alexander Schaefer, Benjamin Acland, Steven Giavasis, Jens Kleesiek, Drew Erickson, René Küttner, Christian Haselgrove, Carlos Correa, Ali Ghayoor, Franz Liem, Jarrod Millman, Daniel Haehn, Jeff Lai, Dale Zhou, Ross Blair, Tristan Glatard, Mandy Renfro, Siqi Liu, Ari E. Kahn, Fernando Pérez-García, William Triplett, Leonie Lampe, Jörg Stadler, Xiang-Zhen Kong, Michael Hallquist, Andrey Chetverikov, John Salvatore, Anne Park, Russell Poldrack, R. Cameron Craddock, Souheil Inati, Oliver Hinds, Gavin Cooper, L. Nathan Perkins, Ana Marina, Aaron Mattfeld, Maxime Noel, Lukas Snoek, K Matsubara, Brian Cheung, Simon Rothmei, Sebastian Urchs, Joke Durnez, Fred Mertz, Daniel Geisler, Andrew Floren, Stephan Gerhard, Paul Sharp, Miguel Molina-Romero, Alejandro Weinstein, William Broderick, Victor Saase, Sami Kristian Andberg, Robbert Harms, Kai Schlamp, Jaime Arias, Dimitri Papadopoulos Orfanos, Claire Tarbert, Arielle Tambini, Alejandro De La Vega, Thomas Nickson, Matthew Brett, Marcel Falkiewicz, Kornelius Podranski, Janosch Linkersdörfer, Guillaume Flandin, Eduard Ort, Dmitry Shachnev, Daniel McNamee, Andrew Davison, Jan Varada, Isaac Schwabacher, John Pellman, Martin Perez-Guevara, Ranjeet Khanuja, Nicolas Pannetier, Conor McDermottroe, and Satrajit Ghosh. Nipype. *Software*, 2018. doi: 10.5281/zenodo.596855.

[6] N. J. Tustison, B. B. Avants, P. A. Cook, Y. Zheng, A. Egan, P. A. Yushkevich, and J. C. Gee. N4itk: Improved n3 bias correction. *IEEE Transactions on Medical Imaging*, 29(6):1310–1320, 2010. ISSN 0278-0062. doi: 10.1109/TMI.2010.2046908.

[7] B.B. Avants, C.L. Epstein, M. Grossman, and J.C. Gee. Symmetric diffeomorphic image registration with cross-correlation: Evaluating automated labeling of elderly and neurodegenerative brain. *Medical Image Analysis*, 12 (1):26–41, 2008. ISSN 1361-8415. doi: 10.1016/j.media.2007.06.004. URL http://www.sciencedirect.com/science/article/pii/S1361841507000606.

[8] Y. Zhang, M. Brady, and S. Smith. Segmentation of brain MR images through a hidden markov random field model and the expectation-maximization algorithm. *IEEE Transactions on Medical Imaging*, 20(1):45–57, 2001. ISSN 0278-0062. doi: 10.1109/42.906424.

[9] Anders M. Dale, Bruce Fischl, and Martin I. Sereno. Cortical surface-based analysis: I. segmentation and surface reconstruction. *NeuroImage*, 9(2):179–194, 1999. ISSN 1053-8119. doi: 10.1006/nimg.1998.0395. URL http://www.sciencedirect.com/science/article/pii/S1053811998903950.

[10] Arno Klein, Satrajit S. Ghosh, Forrest S. Bao, Joachim Giard, Yrjö Häme, Eliezer Stavsky, Noah Lee, Brian Rossa, Martin Reuter, Elias Chaibub Neto, and Anisha Keshavan. Mindboggling morphometry of human brains. *PLOS Computational Biology*, 13(2):e1005350, 2017. ISSN 1553-7358. doi: 10.1371/journal.pcbi.1005350. URL http://journals.plos.org/ploscompbiol/article?id=10.1371/journal.pcbi.1005350.

[11] VS Fonov, AC Evans, RC McKinstry, CR Almli, and DL Collins. Unbiased nonlinear average age-appropriate brain templates from birth to adulthood. *NeuroImage*, 47, Supplement 1:S102, 2009. doi: 10.1016/S1053-8119(09)70884-5.

[12] AC Evans, AL Janke, DL Collins, and S Baillet. Brain templates and atlases. *NeuroImage*, 62(2):911–922, 2012. doi: 10.1016/j.neuroimage.2012.01.024.

[13] Mark Jenkinson, Peter Bannister, Michael Brady, and Stephen Smith. Improved optimization for the robust and accurate linear registration and motion correction of brain images. *NeuroImage*, 17(2):825–841, 2002. ISSN 1053-8119. doi: 10.1006/nimg.2002.1132. URL http://www.sciencedirect.com/science/article/pii/S1053811902911328.

[14] Robert W. Cox and James S. Hyde. Software tools for analysis and visualization of fmri data. *NMR in Biomedicine*, 10(4-5):171–178, 1997. doi: 10.1002/(SICI)1099-1492(199706/08)10:4/5<171::AID-NBM453>3.0.CO;2-L.

[15] Douglas N Greve and Bruce Fischl. Accurate and robust brain image alignment using boundary-based registration. *NeuroImage*, 48(1):63–72, 2009. ISSN 1095-9572. doi: 10.1016/j.neuroimage.2009.06.060.

[16] Jonathan D. Power, Anish Mitra, Timothy O. Laumann, Abraham Z. Snyder, Bradley L. Schlaggar, and Steven E. Petersen. Methods to detect, characterize, and remove motion artifact in resting state fmri. *NeuroImage*, 84 (Supplement C):320–341, 2014. ISSN 1053-8119. doi: 10.1016/j.neuroimage.2013.08.048. URL http://www.sciencedirect.com/science/article/pii/S1053811913009117.

[17] Yashar Behzadi, Khaled Restom, Joy Liau, and Thomas T. Liu. A component based noise correction method (CompCor) for BOLD and perfusion based fmri. *NeuroImage*, 37(1):90–101, 2007. ISSN 1053-8119. doi: 10.1016/j.neuroimage.2007.04.042. URL http://www.sciencedirect.com/science/article/pii/S1053811907003837.

[18] Theodore D. Satterthwaite, Mark A. Elliott, Raphael T. Gerraty, Kosha Ruparel, James Loughead, Monica E. Calkins, Simon B. Eickhoff, Hakon Hakonarson, Ruben C. Gur, Raquel E. Gur, and Daniel H. Wolf. An improved framework for confound regression and filtering for control of motion artifact in the preprocessing of resting-state functional connectivity data. *NeuroImage*, 64(1):240–256, 2013. ISSN 10538119. doi: 10.1016/j.neuroimage.2012.08.052. URL http://linkinghub.elsevier.com/retrieve/pii/S1053811912008609.

[19] Raimon H. R. Pruim, Maarten Mennes, Daan van Rooij, Alberto Llera, Jan K. Buitelaar, and Christian F. Beckmann. Ica-AROMA: A robust ICA-based strategy for removing motion artifacts from fmri data. *NeuroImage*, 112(Supplement C):267–277, 2015. ISSN 1053-8119. doi: 10.1016/j.neuroimage.2015.02.064. URL http://www.sciencedirect.com/science/article/pii/S1053811915001822.

[20] C. Lanczos. Evaluation of noisy data. *Journal of the Society for Industrial and Applied Mathematics Series B Numerical Analysis*, 1(1):76–85, 1964. ISSN 0887-459X. doi: 10.1137/0701007. URL http://epubs.siam.org/doi/10.1137/0701007.

[21] Alexandre Abraham, Fabian Pedregosa, Michael Eickenberg, Philippe Gervais, Andreas Mueller, Jean Kossaifi, Alexandre Gramfort, Bertrand Thirion, and Gael Varoquaux. Machine learning for neuroimaging with scikit-learn. *Frontiers in Neuroinformatics*, 8, 2014. ISSN 1662-5196. doi: 10.3389/fninf.2014.00014. URL https://www.frontiersin.org/articles/10.3389/fninf.2014.00014/full.
